# Peg-in-Hole Assembly Based on Six-Legged Robots with Visual Detecting and Force Sensing

**DOI:** 10.3390/s20102861

**Published:** 2020-05-18

**Authors:** Yinan Zhao, Feng Gao, Yue Zhao, Zhijun Chen

**Affiliations:** State Key Laboratory of Mechanical System and Vibration, School of Mechanical Engineering, Shanghai Jiao Tong University, Shanghai 200240, China; zhaoyinan1990@sjtu.edu.cn (Y.Z.); yue.zhao@sjtu.edu.cn (Y.Z.); ppq67hwuxe@alumni.sjtu.edu.cn (Z.C.)

**Keywords:** six-legged robot, peg-in-hole assembly, trajectory planning, sensor detect, force control

## Abstract

Manipulators with multi degree-of-freedom (DOF) are widely used for the peg-in-hole task. Compared with manipulators, six-legged robots have better mobility performance apart from completing operational tasks. However, there are nearly no previous studies of six-legged robots performing the peg-in-hole task. In this article, a peg-in-hole approach for six-legged robots is studied and experimented with a six-parallel-legged robot. Firstly, we propose a method whereby a vision sensor and a force/torque (F/T) sensor can be used to explore the relative location between the hole and peg. According to the visual information, the robot can approach the hole. Next, based on the force feedback, the robot plans the trajectory in real time to mate the peg and hole. Then, during the insertion, admittance control is implemented to guarantee the smooth insertion. In addition, during the whole assembly process, the peg is held by the gripper and attached to the robot body. Connected to the body, the peg has sufficient workspace and six DOF to perform the assembly task. Finally, experiments were conducted to prove the suitability of the approach.

## 1. Introduction

In the past few years, research on legged robots has received much attention [1,2,3,4]. Legged robots require merely isolated footholds for movement on the ground, which provides better adaptability in rough terrains, in contrast to tracked and wheeled robots [5,6,7]. According to current studies, legged robots can be used for detecting and operating tasks in complex environments. The walking robot SILO6 was designed to detect and locate antipersonnel landmines [8]. ATHLETE, studied by Wilcox et al. [9], emphasized handling cargos and manipulating objects on the moon. HUBO [10] and ATLAS [11] are biped robots that perform door opening manipulations. The robot COMAN applied the visual positioning method to turn circular valves [12]. These studies demonstrate that with their natural advantages legged robots are able to replace manpower to perform different kinds of tasks such as assembly processes. For instance, during the manual assembly process, professional techniques and high labor cost are required. Especially in some hazardous environments, manual assembly becomes more difficult and can even cause accidents. With the development of legged robots, the application of the robots in complex and dangerous scenarios shows that there is also possibility to employ them in assembly tasks.

In the assembly task research field, analyses usually highlight the key point of the assembly process, which is the peg-in-hole problem. Zhang et al. developed a fuzzy force control strategy for the dual peg-hole fitting using an ABB IRB1200 robot to accomplish an automatic fitting process [13]. Tang et al. designed a novel display device to adopt the data demonstrated by human beings. A modified learning from demonstration (LfD) algorithm was applied to a FANUC manipulator to carry out the peg-in-hole procedure [14]. Besides, Park et al. [15] used industrial manipulators to insert square pegs into square holes. Current studies on peg-in-hole tasks mostly focus on industrial robots and mechanical arms. Compared with industrial robots, legged robots possess the advantage of mobility. During operation, a legged robot can move easily to complete the peg-hole assembly process at different positions. In the realm of legged robots, studies mainly include bipedal robots [16,17], quadruped robots [18,19] and hexapod robots [20,21]. Apparently, the hexapod robot, with better stability during walking and operation, is more suitable for automatic assembly. However, there is no relevant research covering specific approaches for the six-legged robot conducting a peg-in-hole assembly task. Hence, it is of value and helpful to establish an approach for the six-legged robot peg-in-hole implementation.

To fulfill the abovementioned task, the relative orientation and position between the hole and peg need to be set up as a priority. Visual sensors are applied for locating the two. Due to the location uncertainties, Pauli et al. [22] used cameras to collect the edges of the object and examine the overall peg-hole insertion procedure. Huang et al. [23] applied a visual high-speed camera and a single eye-in-hand aimed to match the peg and hole. Su et al. [24] proposed a peg-hole insertion tactic without a force sensor. Based on vision guidance and the attractive region Su et al. presented how the peg-hole insertion was accomplished. Wang et al. [25] and Chang et al. [26] applied visual servo devices to perform the puny hole and peg matching process. In addition to this, force/torque (F/T) sensors were utilized to mate the peg and hole. Li et al. [27] presented a compliant strategy following human testing result for the peg-in-hole task, based on force information and the environment constraints. Abdullah et al. [28] developed an algorithm for the peg-hole insertion task on the basis of human operation. During the insertion process the center of the hole was determined by using a force sensor. In [29], the position of the hole was also judged by the measured force caused by the collision of the peg with the hole area. The six-legged robot, by contrast, can approach the hole from a distance to accomplish the peg-hole insertion operation. Therefore, the locating process in this article is divided into two parts. Firstly, the vision sensor preliminarily locates the relative orientation and position between the hole and the peg. Secondly, the robot moves with its legs to approach the hole, and then makes the peg contact the hole. Based on the feedback of a F/T sensor, the relative location between the hole and peg can be more accurately determined. Since force sensing is used to adjust the position error after visual hole location, a low-cost vision sensor is used for detection in this paper.

When the location process is completed, the peg will be inserted into the hole to perform the assembly task. However, in the case of a small gap between the hole and the peg, a force will be caused due to the robot locomotion error and partial occlusion of the hole during the insertion. In order to ensure smooth completion of the assembly task, force control strategies can be applied. At present, there are two common ways of applying force control tactics for a peg-hole insertion process, namely passive force control and active force control. For the strategy of passive control, some compliant mechanical devices are used to assist the peg insertion into the hole. Yun et al. [30] integrated passive compliance with a learning method to accomplish the peg-in-hole procedure. The compliant device consists of torsional springs and dampers. Pitchandi et al. [31] developed a mathematic model of the peg-hole insertion task containing the stiffness and damping factors of the compliance. Influence of damping in the assembly process was analyzed. Baksys et al. [32] applied remote center compliance (RCC) as the passive compliance device, and the mathematic method of the vibratory alignment with the RCC equipment was proposed. However, it is hard to control the passive compliance equipment, and various operation tasks need various devices, which limits the application of passive force control. By contrast, in terms of the active force control, the external force is sensed by the force sensor and the assembly process is completed by the corresponding control strategy. The impedance control scheme is commonly used for industrial robots to implement the peg-hole inserting task. Kim et al. [33] proposed a shape recognition algorithm on the basis of a force sensor, and impedance control was used to provide stable contact between the hole and the peg during the insertion procedure. Lopes et al. [34] presented a strategy involving cooperation of two robotic manipulators: an industry robot and the RCID manipulator. During the peg-in-hole assembly, RCID preserved its small workspace for force and impedance control. Song et al. [35] proposed a complex-shaped peg-in-hole approach on the basis of a guidance algorithm, and in order to attain stable contact movement, the impedance control applying an admittance filter was conducted. According to related studies, multi-DOF manipulators are used for the peg-hole insertion assembly. In this article, the peg is held by a gripper connected to the body of a six-legged robot, not connected to a multi-DOF robot arm. The robot body, serving as an end-effector, adopts admittance control (another form of impedance control) for the insertion assembly. Based on its own mobility characteristics, the robot can approach the holes from different locations. Moreover, the peg has enough space to work and sufficient DOFs to accomplish the assembly requirement by linking with a 6-DOF body.

In this article, we propose an approach for six-legged robots to accomplish the peg-in-hole task and verify the approach with a six-parallel-legged robot. A vision sensor and an F/T sensor are used for locating the hole and admittance control is applied to guarantee smooth assembly completion. The robot adopts a tripod gait during its movement process.

The rest of the article are divided as follows: in Section 2, a six-parallel-legged robot is introduced and the coordinate system is defined; in Section 3, the means of peg-in-hole assembly is detailed described; in Section 4, the experiment is conducted to testify the proposed approach; eventually, in Section 5, the article is concluded.

## 2. Robot System Overview

### 2.1. The Six-Legged Robot

**Figure 1:**
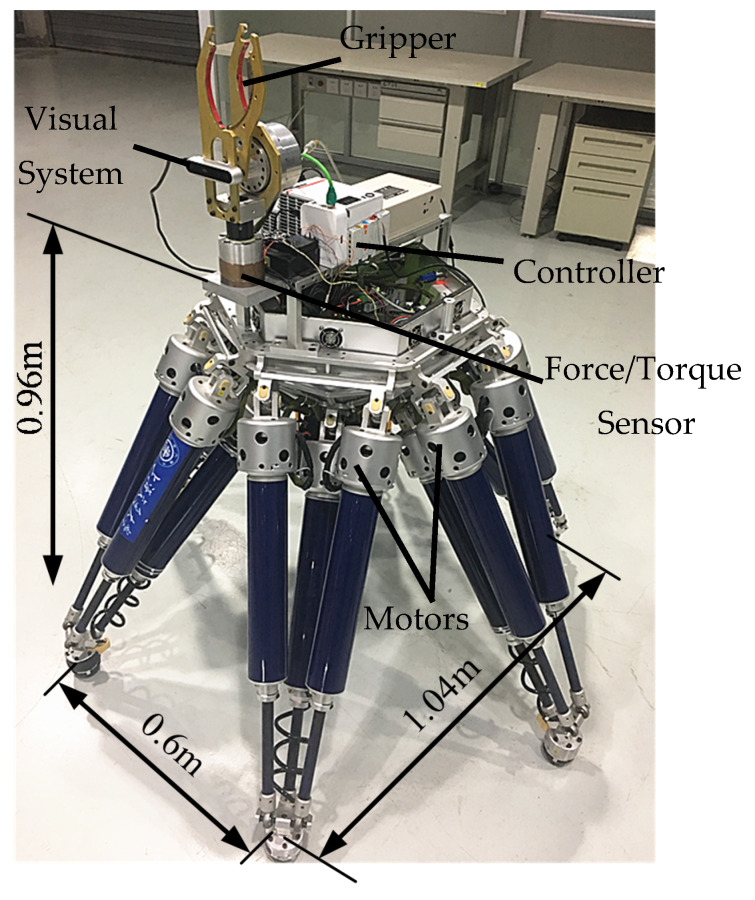
Prototype of the six-parallel-legged robot.

A six-parallel-legged robot named Octopus EDU, illustrated in Figure 1, is a 6-DOF mobile platform with integrated walking and manipulation functionalities. The robot can move on different terrains and be used in complex environments for detection and rescue. A gripper is mounted on the front upper area of the body to enable the robot to complete the peg-in-hole assembly task.

The robot body is a regular hexagon, on which six legs are arranged in diagonally symmetrical. The six legs have an identical mechanical structure, and each of them is a 3-DOF parallel mechanism (UP-2UPS). Benefitting from the 3-DOF, the robot can adapt to different terrains during its movement. Moreover, the parallel mechanism of the legs improves the load capacity of the robot.

**Figure 2:**
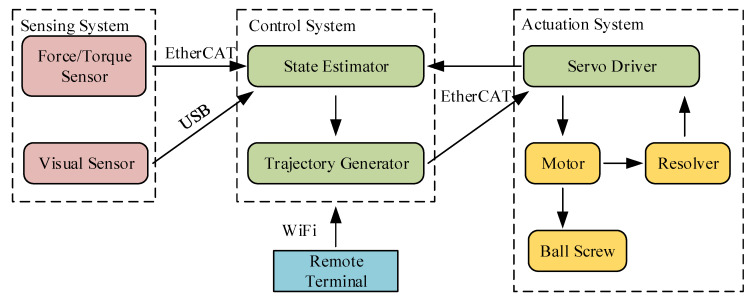
System structure of the robot.

The construction of the robot system is shown in Figure 2. The system includes the sensing, the actuation and the control modules. The sensing system consists of an F/T sensor and a vision sensor. As shown in Figure 1, the F/T sensor is mounted at the end of the gripper and linked to the robot body. The information recorded by the F/T sensor is used to sense external forces. The vision sensor is installed at the front of the body so as to detect visual messages in the moving direction. The vision sensor connects with the control system via USB, and in the peg-in-hole task process, the relative orientation and position between the hole and peg are preliminarily detected by the vision sensor. The actuation system is mainly used to drive the robot. Taking the actuation system of one leg as an example, each leg has three parts and the prismatic joint of the limb is regarded as the active joint. The active joint is actuated by using a servo motor via ball screw linkages. Motors are installed at the top of legs, and each of them is controlled by a corresponding servo driver. The motor has a resolver, by which the real position of the motor can be detected.

The control system, working as the central system of the robot, primarily contains the state estimator and the trajectory generator. Operators can send commands to control the robot via a remote terminal, which is connected to the control system via WiFi. To accomplish the instructions of real-time control, an individual PC running Linux system with Xenomai patch is employed as the control system of the legged robot. Moreover, the control system connects with the actuation system via EtherCAT real-time industrial fieldbus. In this way, the state estimator can supervise the situation of the robot and the trajectory generator can plan motion trajectories in real time. Similarly, the F/T sensor sends the information of external forces to the control system via EtherCAT, so that the robot can adjust the trajectory planning strategy immediately according to the force feedback.

### 2.2. Definition of Coordinate Systems

**Figure 3:**
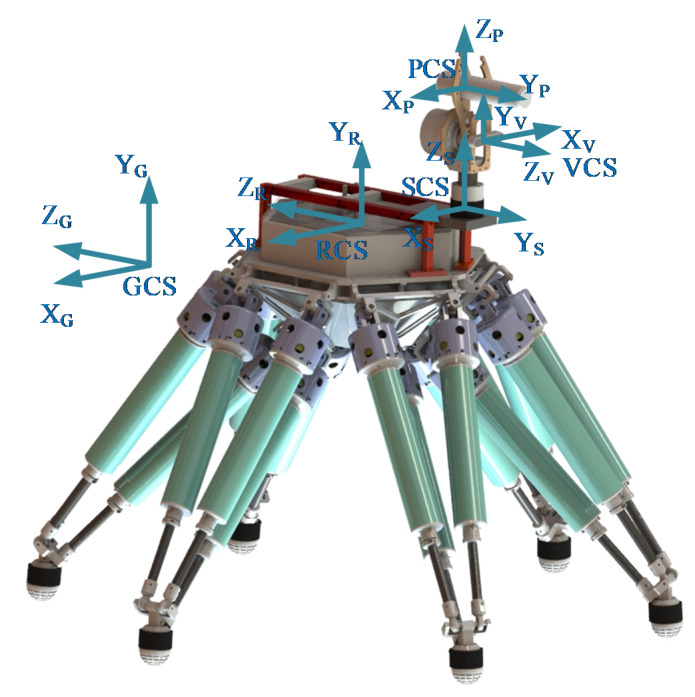
Definition of coordinate systems.

When moving and operating, the motion state of the robot needs to be expressed in various coordinate systems. To guarantee the smoothness of peg-in-hole task, five coordinate systems are established in Figure 3. The first one is the coordinate system of the robot (RCS), the origin of which is fixed in the middle of the robot body. The *Y*-axis and *Z*-axis of the RCS are parallel to the vertical and sagittal axes of the robot, respectively. The location of the RCS changes with the movement of the robot body. The second is the F/T sensor coordinate system (SCS), the origin of which is located at the center of the F/T sensor. The location of the SCS is related to the installation orientation and position of the F/T sensor. For convenience, the directions of the X_S_-axis and the X_R_-axis are consistent, and the same with the Z_S_-axis and the Y_R_-axis. The third is the vision sensor coordinate system (VCS), similar to the SCS, the VCS has been set up after delivery. The VCS location is also related to the mounting position of the vision sensor. The fourth is the peg coordinate system (PCS), which is fixed on the peg. The Y_P_-axis coincides with the center line of the peg and points to the insertion direction during the assembly process. Finally, the ground coordinate system (GCS) is established, which is fixed on the ground and defined by the operator. Since the GCS keeps still when the robot moves and operates, it can refer to the other coordinate systems. For the sake of convenience, the GCS is defined to overlap the RCS if the legged robot is at its original position before the assembly process begins.

Considering that parameters need to be demonstrated in various coordinate systems, the pose matrix **T** is introduced. For instance, TRG indicates the orientation and position of the RCS expressed in the GCS. Similarly, TSR, TVR and TPR describe the orientations and positions of the SCS, the VCS and the PCS in the RCS respectively. As mentioned above, locations of the sensors and the peg determine the pose matrices TSR, TVR and TPR. For instance, the solving process of TSR is stated as below:

(1) TSR=[RSRORS01]

where the matrix RSR indicates the orientation of the SCS in the RCS. ORS is a vector describing the position of the SCS origin expressed in the RCS. For the detailed calculation process readers can refer to [36]. Moreover, TVR and TPR can also be determined by using the same method according to the locations of the vision sensor and the peg.

## 3. Peg-in-Hole Method

Compared with previous methods that rely on manipulators, we use the legged robot to execute the peg-in-hole task in this paper. When the legged robot stands on the ground, the body of the robot has six DOFs in space. Thus, the robot body can be regarded as an end effector. Without additionally installing multi-DOF mechanical arms, the assembly task can be completed by the legged robot itself. The method presented in the paper can enrich the study of legged robots. Moreover, the manipulator usually has a fixed base, whereas the legged robot has the mobility to move to different locations. During the peg-in-hole process, the robot can walk to the hole from a long distance. When the robot reaches the location that the vision sensor can detect the hole, the task can be accomplished through the subsequent method. The legs of the robot are not allowed to slip during the entire task.

**Figure 4:**
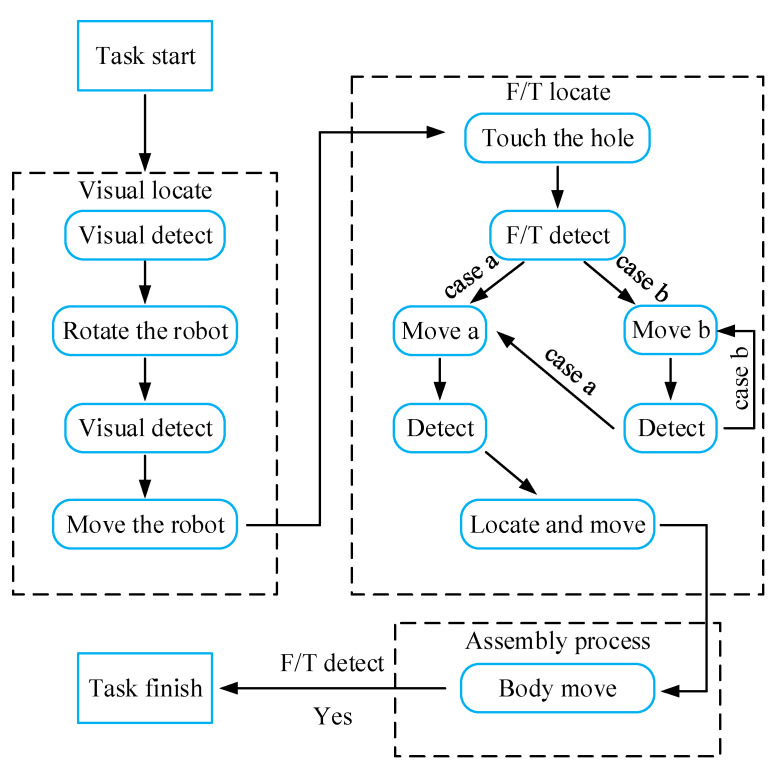
The process of the peg-in-hole task.

The sensing system of the robot consists of the vision sensor and the F/T sensor. The relative location between the hole and peg is determined by the combination of visual and force sensing. And then the control system plans the motion of the robot to proceed the peg-in-hole task. The chamfered peg-hole insertion task is introduced in this article. As exhibited in Figure 4, the whole task includes three subtasks: visual localization, F/T location and the assembly process.

In the process of the visual location subtask, the relative position and orientation between the hole and peg are adjusted preliminarily according to the detection results of the vision sensor. During the F/T location subtask, the peg and hole are located more precisely via the F/T sensor. During locating by the F/T sensor, the peg contacting the chamfer of the hole is defined as Case a, whereas no touching is defined as Case b. Then, the corresponding motions Move a and Move b are applied to adjust the relative position between the hole and peg, respectively. In the end, during the assembly process subtask, the peg is inserted into the hole with the movement of the robot body. During the insertion process, the F/T sensor detect the completion of assembly task. The rest of the paper introduces the three subtasks in details.

### 3.1. Locating the Hole with the Visual System

In the visual location subtask, the relative orientation between the peg and hole is detected by the vision sensor first. Then according to the detection result, the robot rotates to make the peg parallel to the hole. After that, a second visual detection is carried out to modulate the relative location between the peg and hole. The maximum initial error of the approach is mainly limited by the camera view. The initial position for visual detecting allows ±10° rotational error and ±50 cm positional error. Before the visual detection, the robot can move into that visual range from different locations under the user’s remote control.

#### 3.1.1. Visual Detecting Design Scheme

**Figure 5:**
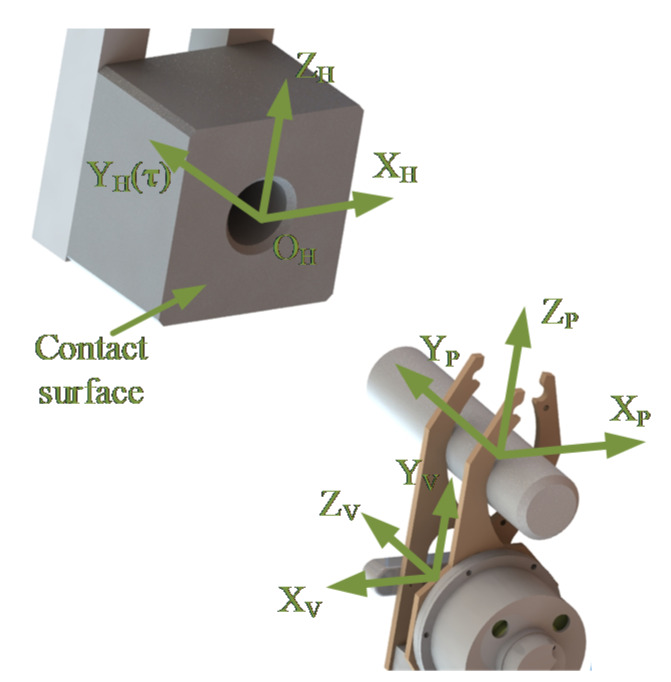
Definition of the HCS.

As exhibited in Figure 5, the hole coordinate system (HCS) is set up, the origin of which is located at the cross point between the axis of the hole and the contact surface. The directions of the Y_H_-axis and the hole-axis are the same. When detecting using the vision sensor, the location of the hole can be determined according to Y_H_ and O_H_. Since the axis of the hole is perpendicular to the contact cover, the direction vector of the hole-axis can be expressed by τ, which is the normal vector of the contact surface.

**Figure 6:**
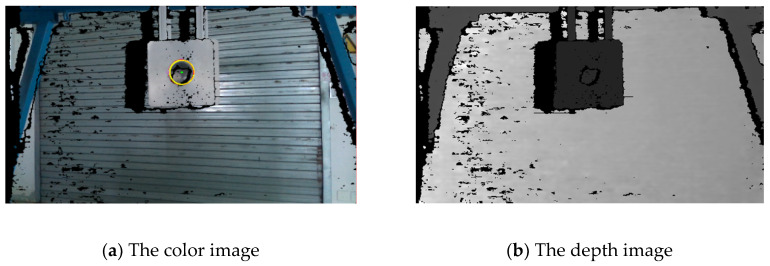
The color image and the depth image.

Color images, depth images and point clouds of objects can be obtained by the vision sensor. Identification of the hole can be separated into two steps. Firstly, the point cloud of the contact surface is extracted from the environment, and the normal vector τ described in the VCS is derived by fitting the point cloud information. Thus, the first step of visual detection is fulfilled. The robot rotates according to the detection result, and proceeds with the second positioning. As shown in Figure 6a, the Hough-transform algorithm is used in color images to obtain pixel positions of the edge of the hole. Then, as exhibited in Figure 6b, these pixels are mapped to the depth image, and marked as the candidate points of the hole on the contact surface. After that, the candidate points in the depth image are mapped into the point clouds. Depending on the change of gradient between the candidate points and the surrounding points, the points of the circular hole on the contact surface is selected. Finally, fitting the selected points, we can obtain the vector OVH, that represents the position of the hole in the VCS. By using the following coordinate transformation, the vectors τ and **O**_H_ can be described in the PCS. Thus, the relative location between the peg and hole is derived:

(2) τP=RPR−1RVRτV

(3) [OPH1]=TPR−1TVR[OVH1]

where the orientation matrices RPR and RVR, denote the orientations of the PCS and the VCS in the RCS, respectively.

#### 3.1.2. Trajectory Planning for Visual Locating

**Figure 7:**
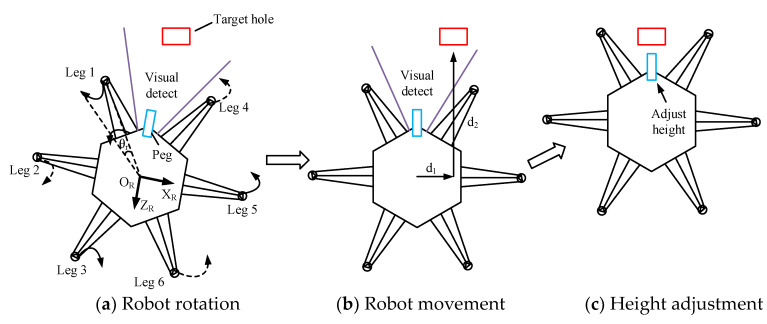
Trajectory planning during the visual locating process.

During the visual location procedure, the trajectory planning of the robot is produced by position control. The trajectory planning can be divided into three parts, as shown in Figure 7. The first part is illustrated in Figure 7a: according to the relative angle $\theta_{r}$ between the peg and hole detected by the vision sensor, the body and the legs move together which enables the robot to rotate an angle $\theta_{r}$. After the rotation of the robot, the vision sensor is used to detect whether the peg is vertical to the cover of the hole. If not, the above motion is repeated until the angle error is eliminated. The second part is described in Figure 7b: the vision sensor detects again to observe the relative position between the hole and peg, and then the robot moves distances d_1_ sideways and d_2_ forward to approach the hole. The third part is exhibited in Figure 7c: the robot adjusts the height of the peg to complete visual location.

As the body and the legs move together in the first and second parts, it is necessary to design the trajectory of the leg. The six-legged robot usually adopts the tripod 3-3 gait, which needs the legs to move alternately for stable walking.

As exhibited in Figure 7a, the robot is at its original position, thus the RCS superposes the GCS. During the rotation process, the robot usually takes one step to rotate to the corresponding position. After the robot rotates $\theta_{r}$ along the Y_G_-axis, the relative position between the body and the leg remains unchanged. Therefore in the first part, the trajectory of leg i in the GCS can be expressed as follows:

(4) EGi=RYG(θr)PGio−PGio

where the position vector PGio represents the initial position of leg i foot-tip, and EGi means the change of the foot-tip position. The trajectory of the leg is generated according to EGi.

In the second part, the movement of the robot is a parallel motion. In Figure 7b, the process by which the robot moves a distance d_1_ along the X_R_-axis is analyzed as an example. Considering d_1_ may be larger than the maximum stride length d_max_, the number of the step and the length of each step need to be calculated in the walking process. When the robot walks, its six legs move alternately in two groups. The first stride length of the first group legs and the last stride length of the second group legs are half of the other stride lengths. Thus the equation that expressing the relationship between the step number and the stride length can be obtained:

(5) $$\left(N-\frac{3}{2}\right)d_{\max}<d_{1}\leq \left(N-\frac{1}{2}\right)d_{\max}$$

where N represents the step number. As N is an integer, we get the value of N. Then we input N into Equation (6) and the stride length d_N_ can be calculated. Thus, the trajectory planning of the second part is also completed.

(6) $$d_{N}=\frac{d_{1}}{N-0.5}$$

### 3.2. Locating the Hole by the F/T Sensor

Via the visual location procedure, the relative location between the peg and hole is located preliminarily. Considering the small clearance between them during the insertion procedure, it is more precise to adopt the F/T sensor to locate the relative location between the hole and peg more precisely.

#### 3.2.1. Positioning Scheme Based on the F/T Sensor

The F/T sensor collects the data of force and torque every millisecond, and the data recorded at millisecond n is expressed as follows:

(7) SSn=[FSnMSn]=[FSnxFSnyFSnzMSnxMSnyMSnz]T

In the above equation, FSn and MSn represent the force date and the torque data, respectively, in the SCS. For convenience, the force and torque data should be expressed in the PCS:

(8) FPn=RSPFSn

(9) MPn=RSP(MSn−OSP×FSn)

where RSP describes the orientation of the SCS in the PCS. OSP is a vector describing the origin of the PCS described in the SCS.

During the F/T location process, the change of the robot motion status will lead to errors when the peg contacts the hole. A follow up procedure is applied to decrease the inaccuracy. When detecting the contact, the robot stops moving and remains stationary for a while. Then the peg leaves the hole. In this process, the recorded forces and torques are used to calculate the more accurate contact data **S**_avg_:

(10) $$S_{\mathrm{avg}}=\left[\begin{array}{c} F_{\mathrm{avg}}\\ M_{\mathrm{avg}} \end{array}\right]=\frac{\sum_{n=n_{0}}^{n_{3}}S_{n}-\sum_{n=n_{0}}^{n_{1}}S_{n}-\sum_{n=n_{2}+1}^{n_{3}}S_{n}}{n_{2}-n_{1}}$$

In the above equation, n_0_ and n_1_ denote the instant the peg contacts the hole and the moment the robot begins to keep still, respectively; n_2_ and n_3_ respectively indicate the moment the robot restarts to move and the instant the peg is completely separated from the hole.

**Figure 8:**
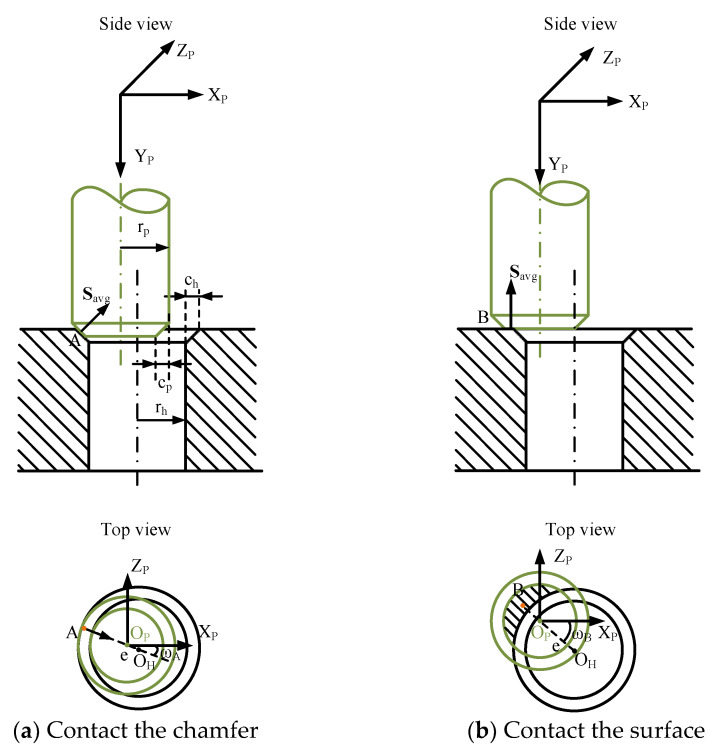
The contact conditions between the peg and hole during the F/T locating process.

After the visual location process, the maximum angle positioning error between the hole and peg is 1º. With this error, the peg can also be inserted into the hole to a certain depth. The angle error can be adjusted during the insertion. Therefore, the force location model focuses on determining the relative position error and is designed under the circumstance that the peg is parallel to the hole ahead of the insertion task. In the F/T location process, based on the possibility of contacting the chamfer, two situations of the model are discussed:

*Case a*. Contact the chamfer of the hole:

The condition of contacting the chamfer of the hole is illustrated in Figure 8a. As shown in the figure, r_p_ is the radius of the peg; c_p_ denotes the width of the peg chamfer; r_h_ is the radius of the hole; c_h_ denotes the width of the hole chamfer; e represents the deviation between the hole and peg. When the peg contacts the chamfer of the hole:

(11) $$r_{p}-c_{p}+e<r_{h}+c_{h}$$

The contact point between the peg and the hole chamfer is defined as A. Since the shapes of the hole and peg are cylindrical, the direction of the contact force coincides with the line O_H_O_P_ in the X_P_O_P_Z_P_ plane, as shown in Figure 8a. The force and torque data, SSn, is recorded by the F/T sensor. And calculating SPn via Equations (8) and (9), the data SPn described in the PCS is obtained. Then SPavg can be derived according to Equation (10). Thus, the angle ω_A_ between the vector **O**_P_**O**_H_ and the X_P_-axis in Case a can be obtained:

(12) {ωA=arctanFPavgzFPavgx+πFPavgx<0ωA=arctanFPavgzFPavgxFPavgx>0

where FPavgx and FPavgz denote the force data along the X_P_-axis and the Z_P_-axis, respectively, in the PCS.

*Case b*. Contact the surface of the hole:

When the value of the deviation e makes Equation (11) invalid, the peg contacts the surface of the hole. As exhibited in Figure 8b, the contact point between the peg and the cover of the hole is defined as B. According to the side view of Figure 8b, ideally the contact force is parallel to the peg-axis. In real tests, friction forces exist. As the friction force is light and the final location is based on Move a, the force along the Y_P_-axis can be extracted for calculation. In this case, FPavgx=FPavgz=MPavgy=0, and torque analyze is the means to solve the position of B in the X_P_O_P_Z_P_ plane:

(13) {MPavgx=−zPBFPavgyMPavgz=xPBFPavgy

where FPavgy is the force data along the Y_P_-axis in the PCS. MPavgx and MPavgz represent the torque data along the X_P_-axis and the Z_P_-axis, respectively, in the PCS. Point B is the centroid of the interface. According to symmetry of the circle, the points B, O_P_ and O_H_ are located in a straight line. Therefore, the angle ω_B_ between the vector **O**_P_**O**_H_ and axis X_P_ in Case b is obtained:

(14) {ωB=arctanzPBxPBxPB<0ωB=arctanzPBxPB+πxPB>0

The relative position between the hole and peg is expressed by the vector **O**_P_**O**_H_, which includes the orientation information ω and the size information e. ω_A_ and ω_B_, the orientation information of **O**_P_**O**_H_ under different contact conditions, are obtained according to the above method. Then, the deviation e can be derived in the process of trajectory planning as follows.

#### 3.2.2. Trajectory Planning for F/T Locating

For trajectory planning, the control system sends the robot’s instantaneous position to the actuation system every millisecond. During the F/T location process, an instantaneous stop is required when the peg contacts the hole. In order to fulfill the above requirement, the gait of the body is planned via a discrete force control approach in this situation:

(15) $$\left\{ \begin{array}{l} F_{\mathrm{mov}}=M_{\mathrm{mov}}A_{n}+C_{\mathrm{mov}}V_{n-1}\\ V_{n}=A_{n}\Delta t+V_{n-1}\\ D_{n}=V_{n}\Delta t+D_{n-1} \end{array}\right.$$

In Equation (15), the 6D vectors **A**_n_,, **V**_n_ and **D**_n_, describe the acceleration, the velocity and the trajectory of the robot body with respect to time n, respectively. △t represents the space of time between two commands that the controller sends. **F**_mov_ is a virtual 6D force vector decided by the moving direction. **M**_mov_ = diag (m_1_, m_2_, …, m_6_) and **C**_mov_ = diag (c_1_, c_2_, …, c_6_) are the mass and damp matrices, respectively.

When the peg contacts the hole, the robot plans the body trajectory by applying different **F**_mov_. **F**_mov_ is a 6D vector, its first three elements describe the change of the position and its second three elements represent the change of orientation. In the process of the actual moving, the contact between the hole and peg shall be judged by **S**_n_, which is the force and torque data recorded by the F/T sensor. If the value of the force feedback exceeds the specific range, it means that the peg touched the hole. At this moment, the elements of **F**_mov_ are all set to zero to achieve the instantaneous stop of the robot. According to Equation (15), the contact force sensed by the F/T sensor is not used for calculation. The contact force has no impact on trajectory generation in Equation (15). Thus, **M**_mov_ and **C**_mov_ are merely determined by the required accelerations and velocities of the body. The acceleration can be changed according to **M**_mov_. And the velocity can be derived from **F**_mov_ and **C**_mov_, when the robot moves at a constant speed. The third term in Equation (15) is used to generate the target positions during the F/T locating process. The trajectory planning method is introduced in detail as follows.

**Figure 9:**
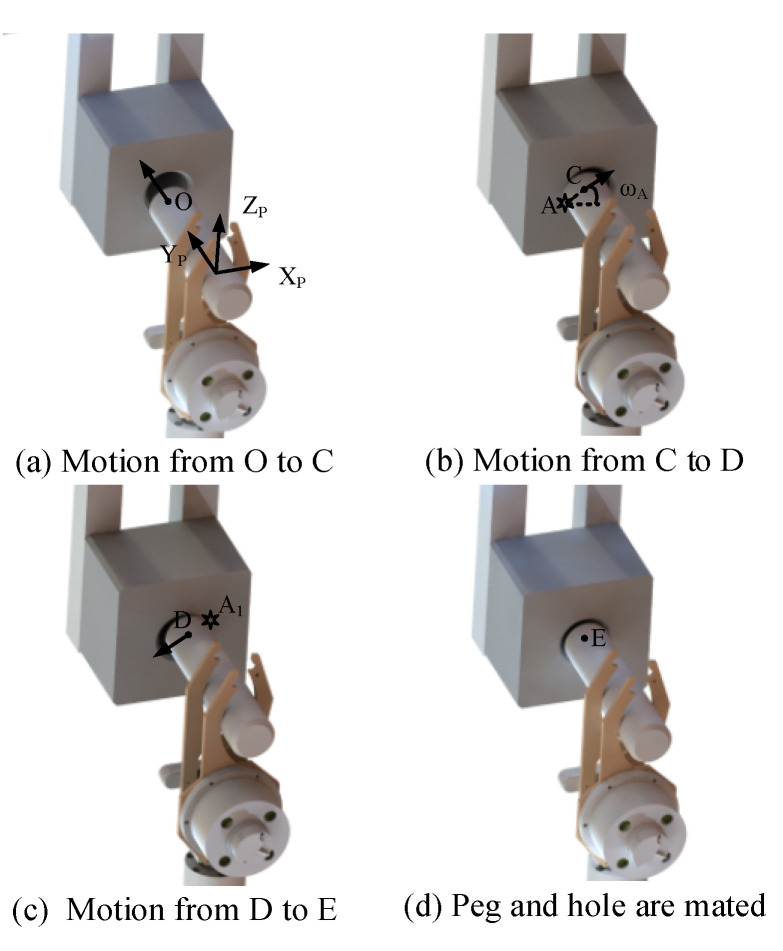
The movement of the peg when the peg contacts the chamfer of the hole.

**Figure 10:**
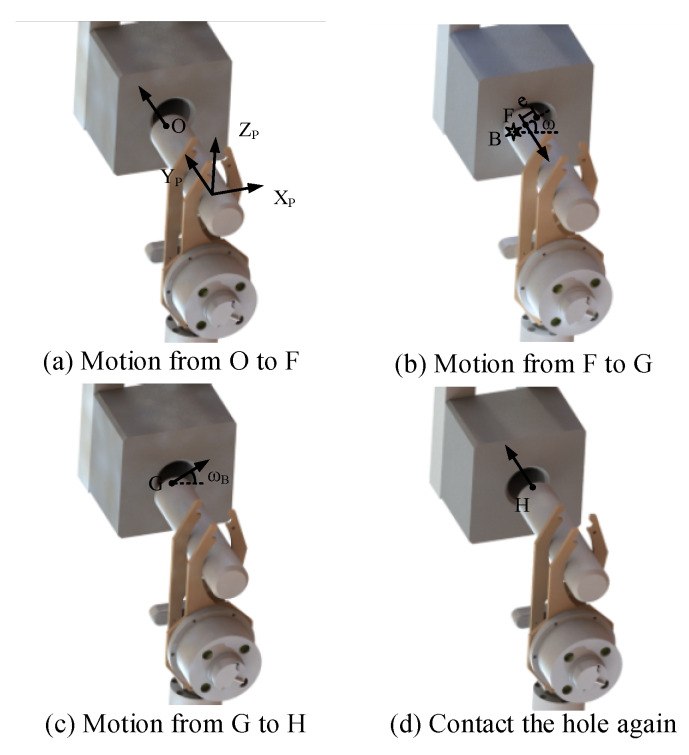
The movement of the peg when the peg contacts the surface of the hole.

Before the peg moves, the peg position is defined as O. The peg moves along the Y_P_-axis to approach the hole at first, as shown in Figure 9a and Figure 10a. Then after contacting the hole, the trajectory of the peg is generated according to the contact condition. If the contact condition is Case a, the robot adopts the motion Move a to adjust the position of the peg. As exhibited in Figure 9b, the peg contacts the chamfer of the hole at point A, and the peg locates at the position C. According to ω_A_ calculated in Equation (12), the movement direction of the peg is obtained. In Figure 9b,c, the peg moves along the direction of the angle ω_A_ until it contacts the other side of the chamfer at point A_1_. At this moment, the end of the peg is located at point D, and the movement that the peg contacting the other side of the chamfer is the motion C→D of the peg. The distance from C to D is defined as d_A_. Since d_A_ = 2e, the deviation e is derived. After that, the peg moves the distance e along the direction from D to C. Finally, the peg moves to the position E to mate the peg and hole. In the above movement, the peg will contact the hole during the motion O→C→D, and the trajectory is planned via Equation (15). The peg moves with the robot body, and the movement is planned in the GCS. Therefore, during the motion O→C→D, **F**_mov_ is generated as follows:

(16) Fmov={(RPG[010]TRPG[000]T)O→C(RPG[cosωA0sinωA]TRPG[000]T)C→D

In the above equation, RPG=RRGRPR. During the motion O→C, the matrix [010]^T^ means the virtual force in the PCS and we can mark it as 1 N along the Y_P_-axis. Meanwhile, **C**_mov_ is set to calculate the velocity of the robot. For instance, the virtual damp factor along the *Y*-axis in the PCS can be set to 50 N·s/m. When the robot moves at a constant speed, the velocity along the Y_P_-axis is 0.02 m/s. When the peg locates at the position C, it will stay static for a while. In this case, all elements of **F**_mov_ are set to zero. During the motion C→D, **F**_mov_ is determined by ω_A_ via Equation (16). **M**_mov_ and **C**_mov_ are deduced from the required acceleration and velocity. We want to obtain a relatively large acceleration, so in the experiment we set **M**_mov_ = diag(1, 1, 1, 1, 1, 1) during the F/T locating process. If a larger acceleration is needed, the values of **M**_mov_ can be reduced. When the robot moves at a constant speed during the F/T location process, we want the total velocity to be 0.02 m/s. Thus, the values of **C**_mov_ can be set to **C**_mov_ = diag(50, 50, 50, 50, 50, 50) in the experiment. According to the values of **C**_mov_, when the robot moves at a constant speed during the motion C→D, the velocities along the X_P_-axis and the Z_P_-axis in the PCS are 0.02cosω_A_ m/s and 0.02sinω_A_ m/s, respectively.

If the contact condition is Case b, the robot applies the motion Move b to adjust the position of the peg. As exhibited in Figure 10a,b, the peg moves from O to F. The peg contacts the surface of the hole and B is the force point of the interface. ω_B_ is calculated according to Equation (13) and Equation (14). Since the peg is pressed on the surface of the hole at the position F, the other motions should be proceeded after the peg moving away from the hole. Thus in Figure 10b,c, the peg leaves the hole along the Y_P_-axis. Then, after moving to the position G, the peg moves the distance d_B_ along the direction of the angle ω_B_. According to Equation (11), if the contact condition is Case b, we can get that e ≥ r_h_ − r_p_ + c_h_ + c_p_. Therefore, in order to decrease the position error between the hole and peg, we can make the distance d_B_ = r_h_ − r_p_ + c_h_ + c_p_. After that, as denoted in Figure 10d, after the motion from G to H, the peg moves along the Y_P_-axis to contact the hole again. And the contact condition is analyzed via the feedback of the F/T sensor. If the contact condition is Case b, repeat the motion Move b. Otherwise, the motion Move a is applied to mate the peg and hole.

The F/T locating process is illustrated in the F/T locate block diagram of Figure 4. The peg and the hole are mated by applying the two motion patterns, Move a and Move b. In the process of Move b, the peg will contact the hole when moving from O to F. During the motion from O to F, the 6D vector can be generated via Equation (17):

(17) Fmov=(RPG[010]TRPG[000]T)O→F

In addition, during the movement F→G→H, the peg will not contact the hole. Thus in this case, position control can be applied to plan the trajectory of the body. Finally, when the peg moves from H to the hole position, the trajectory planning is the same as the motion from O to F.

### 3.3. Assembly Strategy

**Figure 11:**
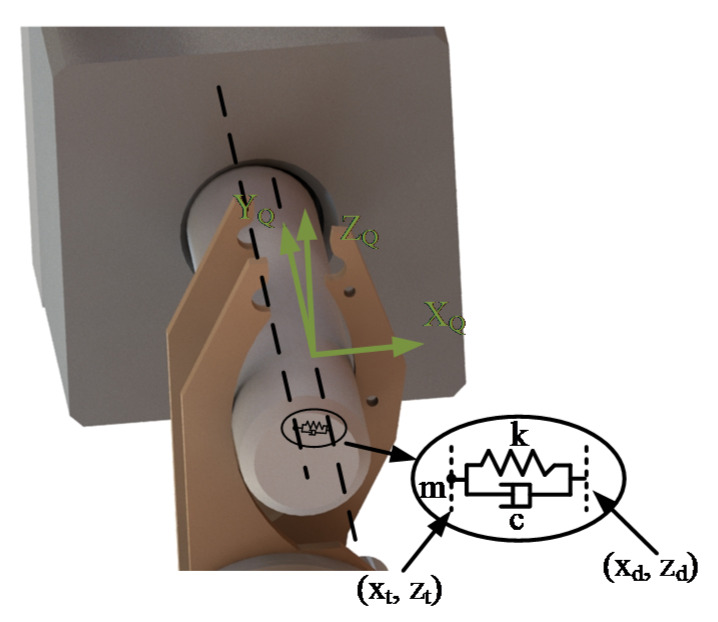
The virtual mass-spring-damper model.

After the location procedures, the peg is inserted into the hole to perform the assembly task. However during the insertion, the movement error of the robot affects the assembly result. When the peg and the hole are mated, a fixed coordinate system (QCS) is set to superpose the current PCS before the insertion process. In the QCS the insertion direction is along the Y_Q_-axis, and the motion along Y_Q_ is planned via Equation (15). Thus, the insertion process can be stopped immediately when the external forces on the peg exceed a certain range. In addition, the peg contacts the wall of the hole along the X_Q_OZ_Q_ plane. In order to guarantee the smooth assembly, the authors present the admittance control. Therefore, the robot body performs as if its target and desired positions were linked via a virtual mass-spring-damper system, as shown in Figure 11.

**Figure 12:**
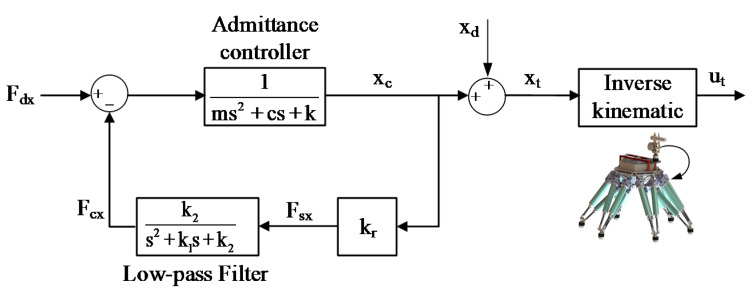
The robot admittance control system.

In Figure 11, (x_d_, z_d_) and (x_t_, z_t_) denote the desired and the target positions in workspace separately. Based on the difference between these two positions, the robot can actively generate the virtual damping and spring forces. In addition, m, c and k represent parameters of the mass, the damping and the stiffness successively. Thus, the trajectory along the X_Q_OZ_Q_ plane can be planned. Figure 12 shows the robot admittance control system.

As exhibited in Figure 12, F_dx_ represents the desired force along the X_Q_-axis. F_sx_ is the force information recorded by the F/T sensor, and the contact force F_cx_ is extracted through the filter. Then, depending on F_dx_ and F_cx_, the compensated position x_c_ along the X_Q_-axis is obtained via the admittance filter. After that, x_t_ can be derived according to x_d_ and x_c_ and z_t_ can also be obtained by applying the same method. Finally, the target position u_t_ in the robot joint space is calculated by using the inverse kinematic model, and thus trajectory planning of the motion is generated. The kinematic model was introduced in [37].

In the above control process, the parameters of the admittance controller and the low-pass filter have a significant influence on the reliability of the system. Analyzing the transfer function of the system, we can obtain:

(18) $$G(s)=\frac{s^{2}+k_{1}s+k_{2}}{ms^{4}+\left(c+mk_{1}\right)s^{3}+\left(k+ck_{1}+mk_{2}\right)s^{2}+\left(kk_{1}+ck_{2}\right)s+\left(kk_{2}+k_{r}k_{2}\right)}$$

According to the Routh–Hurwitz stability criterion, the Routh-Hurwitz Table is established and the first column turns out to be:

(19) $$R_{c}=\left[\begin{array}{l} m\\ c+mk_{1}\\ \frac{c^{2}k_{1}+cmk_{1}^{2}+ck+m^{2}k_{1}k_{2}}{c+mk_{1}}\\ \frac{c^{3}k_{1}k_{2}+c^{2}kk_{1}^{2}+c^{2}mk_{1}^{2}k_{2}+ck^{2}k_{1}+cmkk_{1}^{3}+cm^{2}k_{1}k_{2}^{2}-2cmkk_{1}k_{2}-\left(c^{2}k_{2}+2cmk_{1}k_{2}+m^{2}k_{1}^{2}k_{2}\right)k_{r}}{c^{2}k_{1}+cmk_{1}^{2}+ck+m^{2}k_{1}k_{2}}\\ kk_{2}+k_{2}k_{r} \end{array}\right]$$

To guarantee the stability of the system, all elements of **R**_c_ must be positive. Considering that m, c, k, k_1_, k_2_, k_r_ are all positive, only the fourth element (denoted by E_R4_) may become negative. The parameters k_1_ and k_2_ are determined by the filter and not used to regulate the stability. Therefore, two parameters should be critically analyzed. The first one is k_r_, which is unknown and determined by the materials. It may be very large, causing E_R4_ to negative. The second one is c. Observing E_R4_, it can be found that it is cubic to c and quadratic to m and k, which means c has greater weight on the influence on E_R4_. Moreover, the smaller m is, the higher bandwidth of the system will be. And also, k decides the offset of the peg, which should not be too large. As a result, c becomes a key parameter to regulate the system reliability.

In the experiments, by adjusting the filtering effect, k_1_, k_2_ were set to be 11.25 and 63.33. And m, c, k were tuned to be 12, 2254 and 196. In such case, E_R4_ is positive when k_r_ < 24324. That means the system would be unstable if the contact rigidity between the peg and the hole are larger than 24,324 N/m. If the system becomes unstable because of the large k_r_, the parameter c can be enlarged to enable the system stability again.

In the above procedures (illustrated in Figure 12), the orientations of the QCS and the PCS remain the as the force described in the QCS and the PCS. The model of the mass-spring-damper system can be expressed by:

(20) m[x¨Qtz¨Qt]+c[x˙Qtz˙Qt]+k[xQt−xQdzQt−zQd]=[FPdxFPdz]−[FPcxFPcz]

The target position (x_t_, z_t_) is obtained according to Equation (20). Moreover, y_t_ along the Y_Q_-axis is derived via Equation (15). Thus, the trajectory planning in the QCS is obtained, and converted to the GCS as follows:

(21) [xGtyGtzGt1]=TQG[xQtyQtzQt1]

**Figure 13:**
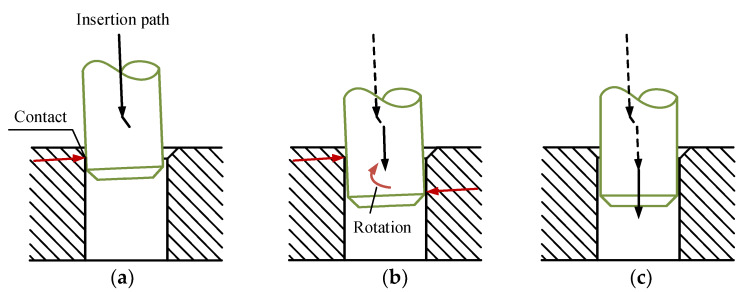
The trajectory planning strategy during the insertion process. (**a**) Insertion with an angle error; (**b**) Compensation for the angle error; (**c**) Insertion after angle compensation.

As mentioned above, the relative location between the peg and hole is compensated by employing the admittance control method. Due to the inaccurate location and the movement during the assembly, the relative angle error between the peg and hole also exists. However, there is a coupling relationship between the position and the orientation during the insertion assembly. Changing both at the same time may affect the smooth completion of the assembly process. In this article, a tactic on the basis of the position admittance control and the orientation compensation is applied to design the trajectory of the peg as exhibited in Figure 13.

Regardless of the angle error, the peg can still be inserted some distance into the hole along the Y_Q_-axis, as denoted in Figure 13a. During the above phase, admittance control is applied to compensate the position error. Then, as shown in Figure 13b, the peg is unable to continue the insertion when contacting two sides of the hole-wall. In this case, the motion generated by admittance control is stopped. During this two-contact phase, the torque applying on the peg is caused by the angular misalignment between the two mating parts. Rotating the peg according to the torque to compensate for the angular error is the process called orientation compensation strategy. And as shown in Figure 13c, after decreasing the angular misalignment, admittance control is used again to continue the assembly process. In addition, according to the new orientation and position of the PCS, we can reset the QCS to coincide with the current PCS.

Therefore, the whole insertion assembly can be regarded as a cyclic process: admittance control → orientation compensation → admittance control. Finally, when the assembly task is finished, the peg is released by the gripper and the robot moves the body away from the peg.

## 4. Experiment and Discussion

### 4.1. Experiment

Experiments were conducted on the six-parallel-legged robot to perform the peg-in-hole assembly and validate the proposed means. During different peg-in-hole manipulations, the robot started to perform the task at different initial positions. The experiments were executed 10 times at each initial position. The success rate was over 80 percent under the summary of the experiments at different initial locations. In the experiment, the diameter of the peg was 50 mm; the clearance between the hole and peg was less than 0.1 mm. Both the peg and the hole were chamfered.

**Figure 14:**
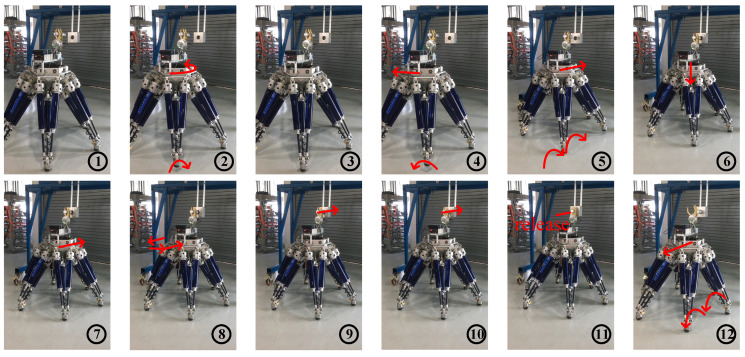
Snapshots of the experiment.

Before the peg-hole insertion, the robot was unaware of the hole location. Once the operation started, the visual sensor was applied to detect the orientation and position of the hole at first. And the visual detection results were applied to guide the robot to walk close to the hole. After that, the robot automatically designed the movement in real time on the basis of the feedback of the force to locate the hole more precisely. Finally, after the alignment procedure, the six-legged robot inserted the peg into the hole. Figure 14 shows snapshots of the experiment, and locations of the robot at diverse instants are stated in subfigures of Figure 14.

Subfigures ① to ⑥ in Figure 14 show the visual locating process. In subfigure ①, the robot was at its initial position and detected the orientation of the hole by the vision sensor. And as shown in subfigure ②, the robot rotated to make the peg parallel to the hole according to the measured orientation information. Then, the robot proceeded the second time visual detection to locate the position of the hole, as denoted in subfigure ③. After that, in subfigures ④, ⑤ and ⑥, the robot approached the hole via lateral (parallel to the X_P_-axis) and forward (parallel to the Y_P_-axis) movements and adjusted the height of the body to mate the peg and hole.

**Figure 15:**
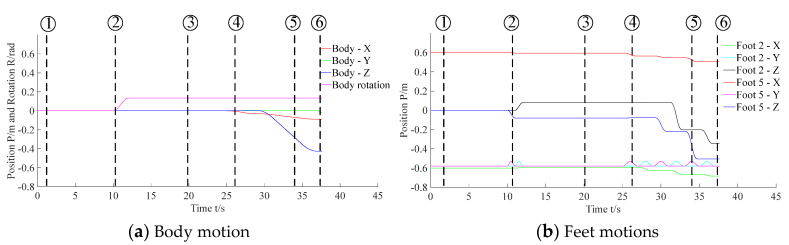
Motions of the body and feet in the process of visual locating.

**Figure 16:**
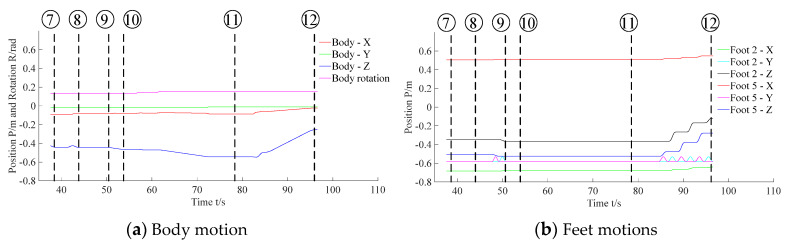
Motions of the body and feet in the processes of F/T locating and insertion assembly.

Figure 15 denotes the body and feet positions during the visual location process. Figure 16 denotes the body and feet positions during the F/T location and insertion assembly processes. Since the peg was held by the gripper and fixed to the body, the trajectory of the peg can be described via the trajectory of the robot body. Besides, as the 2nd and the 5th legs moved alternately in 3-3 gait, here the motions of these two feet were applied to exhibit motions of all feet.

**Figure 17:**
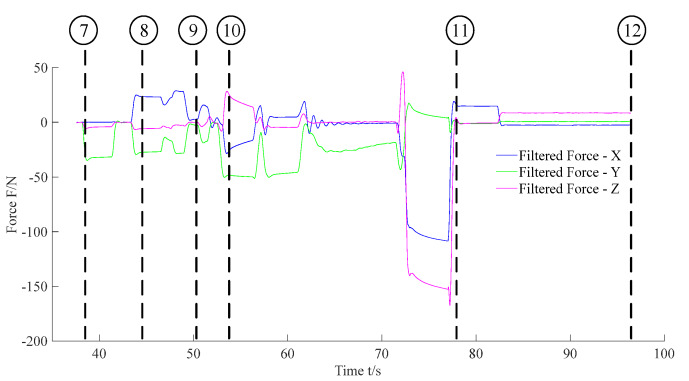
Feedback force during the F/T location and the insertion assembly processes.

**Figure 18:**
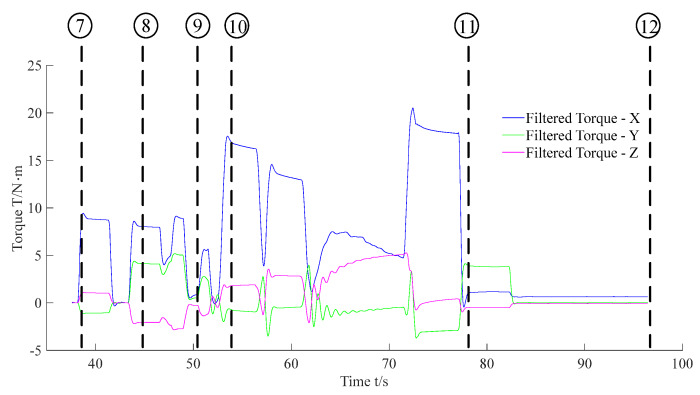
Feedback torque during the F/T location and the insertion assembly processes.

Subfigures ⑦ to ⑫ in Figure 14 show the F/T location and the insertion assembly processes. In addition, Figure 17 and Figure 18 exhibit the data of force and torque recorded in the F/T sensor. As illustrated in subfigure ⑦, the robot body moved forward until the peg contacted the hole. Meanwhile, we can find that force and torque traces illustrated in Figure 17 and Figure 18 change obviously. After ⑦, the traces in Figure 16 illustrate the temporary stationary position of the robot. During the temporary stationary period, the data of F/T sensor was recorded. Using the recorded data, the average contact force/torque can be calculated via Equation (10). And the average contact force/torque expressed in the PCS is derived via Equations (8) and (9). Then, using the method introduced in Section 3.2.1, the robot identified the contact condition of Case b. In this case, the motion, Move b, was applied for another contact. As stated in Section 3.2.2, the motion O→F in the process of Move b is generated via Equation (15), and **F**_mov_ for the motion O→F is calculated via Equation (17). Since the robot rotated in the visual location process before the F/T location process, the matrix RPG used in Equation (16) and Equation (17) should be deduced first. In the experiment, RPG was:

(22) RPG=[0.9951−0.09910001−0.0991−0.99510]

According to RPG and Equation (17), we can obtain that $F_{\mathrm{mov}}={\left[\begin{array}{cccccc} -0.0991 & 0 & -0.9951 & 0 & 0 & 0 \end{array}\right]}^{T}$ during the motion O→F in the experiment.

Then, as shown in subfigure ⑧, the peg contacted the hole again. The traces exhibited in Figure 17 and Figure 18 also change due to the contact. Similar to the previous explanation, the robot judged the contact condition of Case a according to the force feedback. Thus, the motion, Move a, was adopted to align the hole and peg. During the process of Move a, the motion O→C→D is planned via Equation (15).

During the motion O→C, **F**_mov_ is calculated via the first equation in Equation (16). According to RPG in Equation (22), we obtained that $F_{\mathrm{mov}}={\left[\begin{array}{cccccc} -0.0991 & 0 & -0.9951 & 0 & 0 & 0 \end{array}\right]}^{T}$ in the experiment. In addition, during the motion C→D, **F**_mov_ is calculated via the second equation in Equation (16). ω_A_ used in Equation (16) can be calculated via Equation (12). In the experiment, we obtained that cosω_A_ = 0.9723 and sinω_A_ =−0.2337 according to the force feedback. Thus in the experiment, **F**_mov_ was ${\left[\begin{array}{cccccc} 0.9675 & -0.2337 & -0.0964 & 0 & 0 & 0 \end{array}\right]}^{T}$ during the motion C→D via RPG in Equation (22). And the F/T locating process was accomplished after Move a. Then, the robot began to insert the peg into the hole, as shown in subfigures ⑨ and ⑩. Finally, in subfigures ⑪ and ⑫, the peg-in-hole task was finished, and the robot moved away from the hole. Figure 16 denotes the locations of the body and the feet in the processes of F/T locating and the insertion assembly.

### 4.2. Discussion

A six-parallel-legged robot was used in the experiment to demonstrate the methodology presented in the paper. The proposed method can also be used by other six-legged robots to perform the peg-in-hole task considering that six-legged robots with other leg mechanisms can adopt the tripod gait for moving as well. Thus, the legged robot is able to fulfill the peg-hole assembly task by taking advantage of its own mobility without installing additional mechanical arms.

In addition, the focus of this paper is to perform the chamfered peg-in-hole task. There is no change in the visual location process if the method is extended to a chamfer-less situation. The vision sensor is used to detect the hole first, and the robot is adjusted based on the visual feedback. Then, in the following F/T location process, if there is no chamfer, the contact condition is always Case b (defined in Section 3.2.1) when the deviation between the hole and peg is e > e_0_ = r_h_ − r_p_. According to the analysis of Case b introduced in Section 3.2.1, the orientation information of **O**_P_**O**_H_ is derived and the peg moves the distance ne_0_ (n is used to adjust the distance) along **O**_P_**O**_H_ to reduce the deviation. After that, the peg contacts the hole again. If the peg still contacts the same side of the hole according to the force feedback, the peg continues to move along the distance ne_0_ along **O**_P_**O**_H_. If the peg contacts the other side, the new deviation between the hole and peg is ne_0_-e. In this case, the peg moves the distance 0.5ne_0_ along the opposite direction for another contact. The procedure above is repeated until the deviation is smaller than e_0_. Thus, the F/T location process is completed. Finally, the insertion assembly is proceeded. When the clearance between the hole and peg is larger, the insertion process becomes easier due to the larger permissible error. Otherwise, when the clearance is smaller, the insertion speed is reduced to provide more time for adjusting the relative position between the hole and peg.

In the experiments, the F/T location process relied on the results of the visual detection. Occasionally, the orientation information of the vision sensor is not accurate so the experimental results may be affected. To reduce the angle error, the hole is detected 20 times during each visual sensing process and the detection results are averaged to increase the accuracy. Moreover, we give priority to angular precision during the vision location procedure. After the rotation movements, if the angle error is eliminated according to the visual feedback, the robot begins to approach the hole to reduce the position error. Otherwise, the robot continues to rotate to decrease the angle error.

In future work, we plan to improve the presented method in the paper by reducing the reliance on visual detection and various peg-hole forms, such as different sizes and the chamfer-less situation will be studied. In addition, the forces exerted by the legged-robot during the insertion process are related to the frictional interaction between the feet and the ground and the robot needs to maintain its stability during the entire task. As these two challenges are easily satisfied in our case, we did not analyze them concretely. Therefore, we also plan to analyze the two challenges in the case of a low-friction surface with a lighter robot in future.

## 5. Conclusions

The paper proposes a means for a six-legged robot to accomplish a peg-hole insertion task. The conclusions can be summarized as follows:

(1) A vision sensor and an F/T sensor are used to detect the orientation and position of the hole.
(2) On the base of the feedback of the force sensing, the trajectory of the robot is planned in real time.
(3) The peg is held by the gripper and connected to the robot body directly. The body adopts admittance control for the insertion process.
(4) The proposed method is conducted by a six-parallel-legged robot. Based on the mobile performance of the prototype, it can approach holes located at different positions to perform the assembly task.
(5) Verification experiments were conducted, and the experimental results proved the effectiveness of the method.
